# Monomeric streptavidin phage display allows efficient immobilization of bacteriophages on magnetic particles for the capture, separation, and detection of bacteria

**DOI:** 10.1038/s41598-023-42626-9

**Published:** 2023-09-27

**Authors:** Caitlin M. Carmody, Sam R. Nugen

**Affiliations:** Department of Food Science, Cornell University, Ithaca, NY 14853 USA

**Keywords:** Assay systems, Biomaterials, Biotechnology, Nanobiotechnology, Biosensors

## Abstract

Immobilization of bacteriophages onto solid supports such as magnetic particles has demonstrated ultralow detection limits as biosensors for the separation and detection of their host bacteria. While the potential impact of magnetized phages is high, the current methods of immobilization are either weak, costly, inefficient, or laborious making them less viable for commercialization. In order to bridge this gap, we have developed a highly efficient, site-specific, and low-cost method to immobilize bacteriophages onto solid supports. While streptavidin–biotin represents an ideal conjugation method, the functionalization of magnetic particles with streptavidin requires square meters of coverage and therefore is not amenable to a low-cost assay. Here, we genetically engineered bacteriophages to allow synthesis of a monomeric streptavidin during infection of the bacterial host. The monomeric streptavidin was fused to a capsid protein (Hoc) to allow site-specific self-assembly of up to 155 fusion proteins per capsid. Biotin coated magnetic nanoparticles were functionalized with mSA-Hoc T4 phage demonstrated in an *E. coli* detection assay with a limit of detection of < 10 CFU in 100 mLs of water. This work highlights the creation of genetically modified bacteriophages with a novel capsid modification, expanding the potential for bacteriophage functionalized biotechnologies.

## Introduction

Bacteriophages (phages) are viruses that bind and infect specific bacterial strains. Their ability to specifically target bacteria has made them useful tools for detecting and combating their host bacteria^1–5^. Phages have been immobilized to various materials to impart functional capabilities including antimicrobial activity^6,7^, bacteria capture^8,9^, and nanostructure scaffolding^10–12^. T4 phages have been immobilized to polycaprolactone film to functionalize the film with antimicrobial activity resulting in a significant reduction of *E. coli* O157:H7 on beef when in contact with the film^13^. Filamentous phage E2 was immobilized to magnetoelastic filter elements used to capture greater than 90% of target bacteria pathogens passed through the liquid filter system^14^. Additionally, the repeating highly ordered structure of M13 bacteriophages have been utilized to assemble nanowires via polyelectrolyte multilayering for use in a lithium ion battery electrode^15^.

The phages’ proteinaceous structure allows for immobilization via affinity interactions between functional groups on amino acid side chains and material surfaces. A comprehensive collection of phage immobilization strategies can be found in recent reviews^16,17^. For most applications, phages need to be immobilized by their negatively charged capsids to allow proper orientation of the positively charged tail fibers. The tail fibers are responsible for binding bacteria and triggering infection, outward towards the sample. Silica^18,19^, cellulose^20,21^, gold^8,22,23^, and carbon^1,24^ materials have been functionalized with positively charged compounds or polymers to allow immobilization of phages via their capsid. Charge-oriented phages immobilized to chemically functionalized gold have been shown to have higher phage depositing density and bacteria capturing capacity when compared to non-functionalized controls^25,26^. The process of chemically functionalizing materials typically requires toxic organic solvents, complex reactor setups, and expensive equipment^27–29^. Additionally, the effectiveness of charge-based immobilization is often dependent on the ion concentration of the sample matrix.

Alternatively, phages can be genetically modified for site-specific immobilization. A gene for a protein affinity tag fused to a phage capsid protein gene can be incorporated into phage genomes or expressed exogenously from a plasmid to create a capsid modified phage. This well-established “Phage Display” method is frequently used to study protein–protein interactions for pharmaceutical applications^30–32^ and has been employed to immobilize phages to cellulose and streptavidin supports^33–36^. CRISPR/Cas9, can be used to help expedite the editing and selection process for genomic modification of phages^37–39^. This system is composed of a guide RNA (gRNA) which can be designed to target a specific DNA sequence and an endonuclease (Cas9) responsible for double stranded DNA cleavage of the target. This double strand DNA cleavage activates genomic repair pathways which can facilitate of incorporation of new DNA into the genome via homologous recombination^40^. This method has been used in previous work on the same phage and capsid gene that was modified in this study to achieve > 99% recombinant phages produced^41^.

If bacteriophages are to be immobilized on magnetic particles for the capture and separation of bacteria, the avidity between the phage and particle must allow for the pulling of a relatively larger bacterium. Streptavidin-modified particles have often been used for separation following binding of a biotinylated recognition element. While the kd of streptavidin–biotin is known to be ≈ 10^−5^ nM, the purified protein is relatively expensive. In the case of magnetic nanoparticles, the required surface functionalization could be several m^2^. ^42^. For the system to have commercial viability, the conjugation method must be cost-effective and scalable.

We hypothesize that a specific, stable, and high affinity monomeric version of streptavidin (mSA)^43^ can be displayed on the surface of bacteriophages using genetic engineering. In this study, the gene for mSA was fused to the highly antigenic outer capsid protein (Hoc) gene in a T4 phage genome to generate a novel capsid modified phage capable of oriented immobilization to biotin coated materials. This allows the more expensive constituent in the streptavidin–biotin system to be synthesized during the infection of host bacteria. The mSA-Hoc then self-assembles on the phage capsid during viral assembly. A luciferase gene carrying variant of the well-characterized, *E. coli* infecting T4 phage was selected for this study due to previous success with genetic modifications^41^ and ability for small and large foreign proteins to be displayed on the capsid while maintaining infectivity^34,44–47^. The functionality of mSA-Hoc was assessed via phage immobilization onto biotin coated magnetic nanoparticles. The ability of the phage-magnetic particle system was demonstrated in a detection assay for *E. coli* from water with a limit of detection of < 10 CFU/100 mLs. The improved conjugation method and demonstrated performance represent a step forward for commercialization of magnetic phage-based sensors for capture, separation, and detection of their host bacteria. This novel phage capsid modification can be extended to other phages to allow for site-specific immobilization to the plethora of widely available biotinylated materials, expanding potential of phage-based detection, biocontrol, and biomedicine technologies.

## Materials and methods

### Bacteria, phage, and plasmids

*E. coli* NEB® 5-alpha chemically competent cells were used for cloning and obtained from NEB (Ipswich, MA, USA). *E. coli* (ECOR #13), a strain isolated from a healthy human, was obtained from the Thomas S. Whittam STEC Center (East Lansing, MI, USA). *E. coli* DH5α and wild type T4 phages were obtained from ATTC (Manassas, VA USA). T4 phages containing NanoLuc:CBM luciferase reporter (NRGP17) were engineered in a previous study^41^. Addgene plasmids pCRISPR (#42875) and pCas9 (#42876) were gifts from Luciano Marraffini^48^. Addgene plasmid pRSET-msa (#39860) was a gift from Sheldon Park.43 Bacteria overnight cultures were grown shaking 90 rpm at 37 °C overnight in Luria–Bertani (LB) broth with appropriate antibiotic supplementation (50 µg/mL Kanamycin for pCRISPR, 25 µg/mL Chloramphenicol for pCas9). Phage propagation via liquid lysate was carried out as described by Bonilla et al.^49^. Phages were initially purified by centrifugation at 3260 × g for 30 min to remove cell debris followed by filtration through a 0.2 µm cellulose nitrate or cellulose acetate vacuum filter system from Corning (Corning, NY, USA) to remove NanoLuc:CBM background. Phages were concentrated via centrifugation (25,000 × g, 3 h, 4 °C) and resuspension in a 0.5 mL of SM buffer. Concentrated phages were incubated with 6 mm cellulose fiber discs from Whatman (Florham Park, NJ, USA) overnight to further remove NanoLuc:Cbm background. Phage enumeration via double layer plaque assay was used to determine Plaque Forming Units (PFU).

### Materials and reagents

All cloning reagents were purchased from New England Biolabs (Ipswich, MA, USA). Nano-Glo luminescent reagent was purchased from Promega (Madison, WI, USA) and prepared immediately before use according to the manufacturer’s recommendations. Luminescent signals were monitored using a Synergy Neo 2 Hybrid Multimode Reader (Biotek Instruments, Winooski, VT, USA). Ninety-six well filter plates (0.2 µm PVDF) were purchased from Corning (Corning, NY, USA). Multi-well plate manifold for filtering 96-well plates was purchased from Pall (Cortland, NY, USA). All other reagents were purchased from Thermo Fisher Scientific (Waltham, MA, USA). Primers are listed in Table S1.

### Donor plasmid construction

The guide RNAs (gRNA) used to target hoc were designed in Geneious Prime (Biomatters, Ltd.,Auckland, NZ) and ordered from IDT as single stranded DNA oligos (Coralville, IA, USA). The gRNA were cloned into pCRISPR (a gRNA expression plasmid for targeting a specific sequence) following Marraffini’s protocol^48^. The donor DNA expression cassette consisting of a chimeric monomer of streptavidin and rhizavidin (mSA) optimized for high-affinity binding biotin^43^, T4 phage hoc codon optimized to be resistant to the gRNA used in this study, and regions of homology to T4 phage hoc were synthesized via PCR amplification using pRSET-msa and T4 phage genomic DNA as templates (Fig. S2). The donor DNA expression cassette constituents and gRNA containing pCRISPR were ligated together via Gibson assembly cloning^50^ following the NEBuilder Hifi DNA Assembly manufacture protocol. The donor plasmid sequence was confirmed via colony PCR in-house followed by Sanger sequencing performed by the Biotechnology Resource Center (BRC) Genomics Facility (RRID:SCR_021727) at Cornell Institute of Biotechnology (Ithaca, NY, USA) using Applied Biosystems Automated 3730xl DNA Analyzers, Big Dye Terminator chemistry, and AmpliTaq FSDNA Polymerase.

### Recombinant phage construction

CRISPR/Cas9 mediated engineering was used to construct recombinant phages as previously described^41^. The system relies on a gRNA designed to target a specific DNA sequence and an endonuclease to generate a double strand DNA break in the target sequence. In the base phage NRGP17, there was a nonsense mutation in hoc approximately 1000 bp away from the site of the desired mSA addition. The distance between these two sites was too large to simultaneously edit both areas at once. Two separate engineering steps were needed with different gRNA for the two modifications. Phage genomes were edited using a modified protocol from Duong et. al.^41^. The phage with the repaired hoc nonsense mutation was named NRGP28. The phage with the mSA-modified Hoc was named NRGP56.

*Creating T4 Recombinants*. *E. coli* NEB 5-alpha cells containing pCRISPR (gRNA + donor sequence) and pCas9 were added to 0.8% LB top agar containing the appropriate antibiotics and mixed. Then 100 µL of 106 PFU/ml NRGP17 phages were added to the same tube containing molten agar, mixed, and poured onto an LB plate. Plates were incubated overnight at 37 °C.

#### Background removal

A spot over assay was done via plaque transfer to minimize endogenous background from the donor plasmid containing *E. coli*. Overnight culture (200 µL) of *E. coli* DH5α without any plasmids was added to molten 0.8% LB top agar, mixed, and poured onto a gridline square LB plate. A sterile toothpick was used to transfer plaques resulting from the previous day’s plaque assay over to the gridline square spot assay plate. Plates were incubated overnight at 37 °C (Supplementary Figure S1).

#### Screening for T4 recombinants

PCR was performed on the plaques following Novagen’s T7Select System’s plaque PCR protocol (Madison, WI, USA). A single plaque PCR confirmed positive phage was propagated, genomes were extracted from lysate using Norgen Phage DNA Isolation Kit (Norgen Biotek Corporation, Canada), and whole genome sequenced performed by the Cornell university College of Veterinary Medicine Animal Health Diagnostic Center, Department of Molecular Diagnostics (Ithaca, NY, USA) via Illumina mySeq platform and Illumina Basespace Sequence Hub for data acquisition and quality control analysis. All sequencing data were analyzed in Geneious Prime® (Biomatters, Ltd., Auckland, NZ).

### Immobilization of phages to biotin coated magnetic nanoparticles

The mSA-Hoc phages were partially pre-blocked with biotin at room temperature for 40 min to help limit crosslinking of the beads using a ratio of 10 biotin molecules to 1 mSA-Hoc protein. Magnetic biotin coated nanoparticles from Raybiotech (Peachtree Corners, GA, USA) were removed from their storage solution and prepared according to the manufacturer’s manual with a modification to the wash buffer (TTBS with 0.5% tween 20 and biotin). Pre-blocked phages (3.5 × 107 PFU) were added to prepared beads and incubated with rotation for 5 min at room temperature. A magnetic stand was used to separate the nanoparticles from the supernatant. Nanoparticles were washed twice with 1 mL wash buffer before final resuspension in 500 µL of wash buffer and stored at 4 °C.

### Detection of *E. coli* in 10 ml

The procedure for detection of *E. coli* in small volume samples (10 mL) has been described previously^51^. Single colonies of *E. coli* (ECOR#13) were grown shaking for 16–18 h in 10 mL of LB broth. Serial dilutions were performed in Phosphate Buffered Saline (PBS). Bacteria were enumerated using standard plate-counting to determine Colony Forming Units (CFU/mL). Samples (10 mL) of autoclaved tap water (Cornell University water supply) were spiked with either 100 µL 10^−7^ *E. coli* dilution (66–124 CFU), 100 µL of the 10^−8^ *E. coli* dilution (3–16 CFU), or l00 µL of PBS. The *E. coli* concentrations of the final inoculum was confirmed using standard plate counts performed in technical triplicate for each biological replicate. A total of 500 µL of 20 × LB broth was added to each sample and were incubated with shaking 200 rpm at 37 °C for 3 h. Phage bead supernatant was removed and replaced with an equivalent volume of LB immediately before use in the detection assay. A total of 250 µL of phage immobilized particles was added to each sample and incubated shaking 90 rpm for 10 min. Samples were poured into square Petri dishes to maximize the surface area and placed against a magnetic rig (magnetic bars adhered to 10 cm^2^ supports). Supernatants were aspirated and discarded followed by particle resuspension in 500 µL of LB broth. Resuspended beads were incubated shaking (90 rpm) for 3 h at 37 °C followed by filtration through 96-well filter plates with a 6 mm cellulose fiber disk added to each well to capture the Nanoluc:CBM. A 50 µL aliquot of NanoGlo reagent was added to each well and let incubate at room temperature for 10 min before reading luminescence. The detection assay was performed using three biological replicates. The Limit of Detection (LOD) was determined using the 0 + 3SD method where the lowest positive signal is defined as the values of the negative control + three standard deviations^52–55^.

### Detection of *E. coli* in 100 ml

Based on previously described protocol^51^. *E. coli* (ECOR#13) preparation and dilution were the same as described above. Bacteria were enumerated using standard plate-counting to determine Colony Forming Units (CFU). Samples (100 mL) of autoclaved tap water were spiked with either 100 µL of 10^−7^ *E. coli* dilution (81 ± 24 CFU), 100 µL of 10^−8^ *E. coli* dilution (9 ± 4 CFU), or l00 µL of PBS (Negative control). Each sample received 5 mL of 20 × LB broth for resuscitation followed by incubation with shaking (200 rpm at 37 °C for 3 h). Phage bead supernatant was removed and replaced with an equivalent volume of LB immediately before use in the detection assay. A total of 500 µL of phage immobilized particles was added to each sample and incubated shaking 90 rpm for 10 min. Samples were poured into square Petri dishes to maximize the surface area and placed against a magnetic rig (magnetic bars adhered to 10 cm^2^ supports). Supernatants were aspirated and discarded followed by particle resuspension in 500 µL of LB broth. Resuspended beads were incubated shaking 90 rpm for an additional 3 h at 37 °C followed by filtration, luminescent detection, LOD determination as described above. The detection assay was performed in three biological replicates.

## Results and discussion

### Genetic engineering of bacteriophages

**Figure 1:**
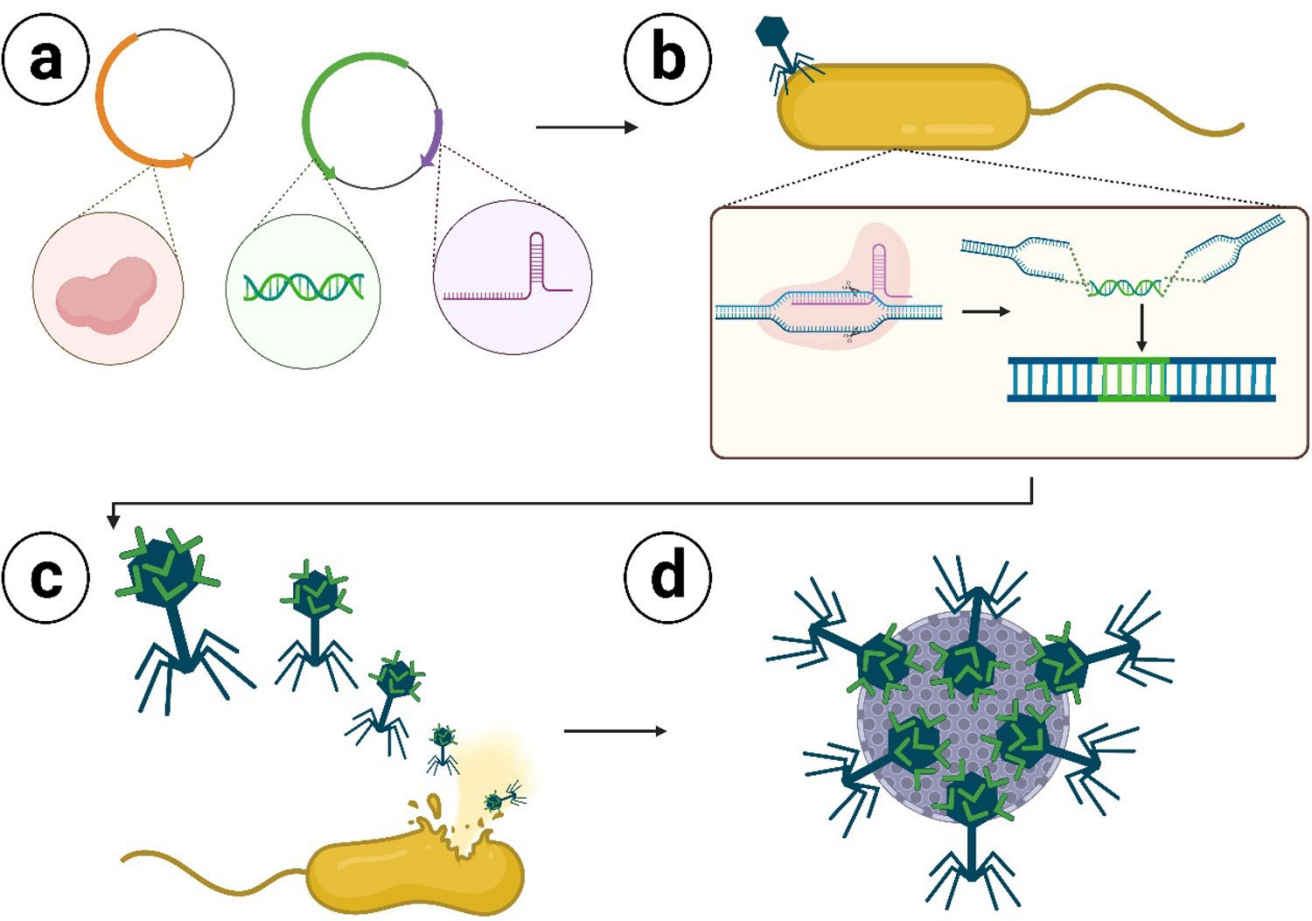
CRISPR/Cas9 genetic engineering of bacteriophages workflow. (**a**) Two plasmids encode all components for forming a CRISPR effector complex able to identify and cleave the targeted DNA sequence in the phage genome. One plasmid also delivers the donor DNA sequence composed of the desired DNA to be incorporated into the phage genome flanked by regions of homology to the phage genome cleavage site. (**b**) Wild type bacteriophages infect a bacteria cell containing the plasmids encoding the CRISPR effector complex components and donor DNA. The phage genome is cleaved by the CRISPR effector complex. The cleaved phage genome repairs itself with the donor DNA sequence via homologous recombination. (**c**) Recombinant phage progeny are released from the bacteria cell at the end of the lytic replication cycle. (**d**) Recombinant phages have improved functional capabilities for numerous biotechnology applications.

The genetic engineering tool, CRISPR/Cas9, was used to translationally fuse a monomer streptavidin affinity tag (mSA) to the nonessential highly antigenic outer capsid protein (hoc) of T4 bacteriophage. The overall genetic engineering process is depicted in Fig. 1. A donor plasmid was synthesized via Gibson Assembly cloning^50^ to incorporate the donor DNA fragment composed of mSA-hoc codon optimized to be resistant to the gRNA targeting hoc flanked by homologous arms (~ 1500 bp). The N-terminus of Hoc was selected for mSA fusion due previous studies demonstrating improved capsid binding of Hoc N-terminal fusion proteins when compared to C-terminus fusion proteins, due to the C-terminus of Hoc containing the T4 capsid protein binding domain^47,56,57^. Large homologous arm lengths were chosen because it has been reported that increasing homologous arm length can increase recombination rate^37,58^. Within the bacteria cell, the CRISPR effector complex facilitated the recombination of wild type T4 phage with the donor DNA to produce recombinant mSA-hoc phage progeny (NRGP56). Phage genomic incorporation of mSA was confirmed via Sanger and whole genome sequencing (Supplementary Information 2) of plaques.

### Immobilization of engineered bacteriophages

**Figure 2:**
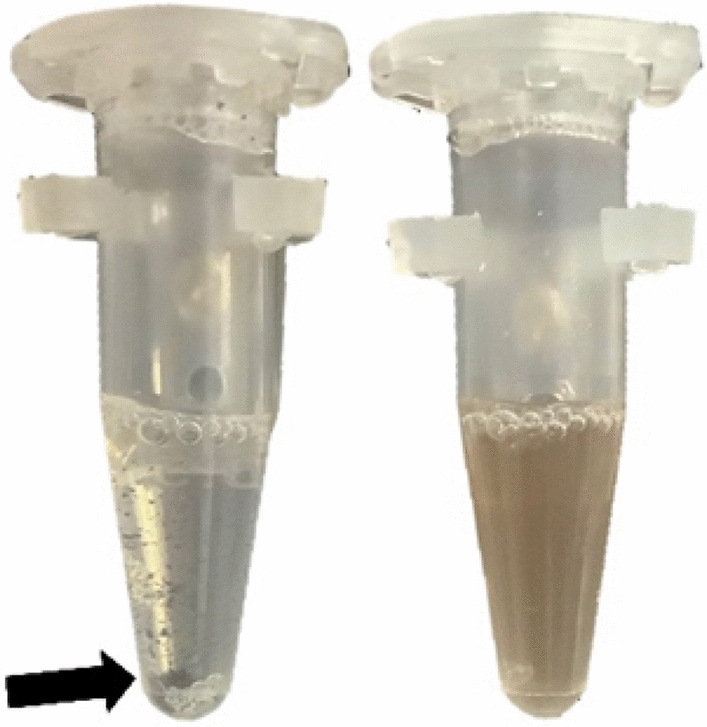
Crosslinked NRGP56 phage nanoparticles. Tubes contained the same titer of NRGP56 (left) or NRGP28 (right) phages incubated rotating for one hour on a circular rotator at room temperature. Left tube contains NRGP56 phages that resulted in nanoparticle crosslinking of the particles and loss of suspension. The black arrow indicates the aggregated phage/nanoparticles. The right tube contains NRGP28 phages with no indication of crosslinking or loss of suspension.

The NRGP56 phages were immobilized to biotin coated magnetic nanoparticles to assess the ability of the mSA affinity tag to facilitate immobilization. While the concentrations of modified Hoc was not sufficient for an ELIZA or Western blots, when the mSA-modified NRGP56 phages were mixed with the biotin-modified magnetic particles, significant crosslinking between phages and particles resulted in the aggregation and settling of the phage-particle mass, a phenomenon not observed with the NRGP28 phages (Fig. 2). To mitigate crosslinking of nanoparticles seen during early phage immobilization phages were initially incubated with free biotin to block some of the available biotin binding sites. Then NRGP56 phages were incubated with biotin coated magnetic nanoparticles with a diameter of 500 nm alongside NRGP28 (wild type Hoc) phages as a control. The phage bead solutions were washed with wash buffer to remove unbound phages. Titering of the washes revealed the majority of the NRGp56 and NRGP28 phages remained immobilized on the beads (data not shown). The control phages remaining on the beads is likely due to weak electrostatic adsorption, which can be affected by changes in ionic strength, temperature, shear force, and pH^59–61^. Therefore, this interaction may not be sufficient for keeping phages immobilized in some applications. To test the practical significance of modifying Hoc for immobilization, NRGp56 and NRGP28 phages immobilized to nanoparticles were compared in an *E. coli* detection assay.

### *E. coli* detection assays

**Figure 3:**
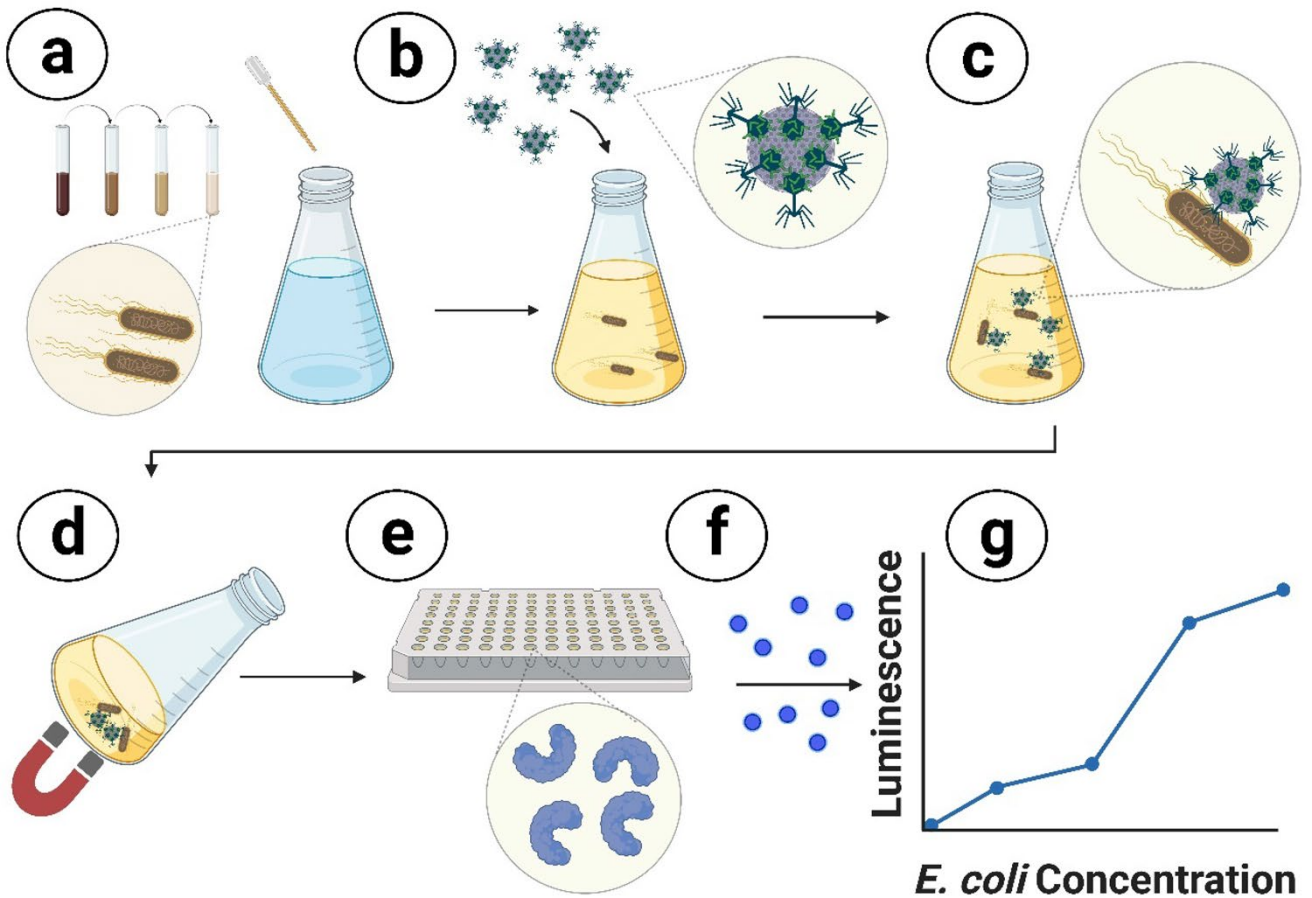
Bacteriophage-based *E. coli* detection assay workflow. (**a**) Sterile tap water samples were spiked with varying concentrations of *E. coli* and incubated shaking at 37 °C for 3 h to resuscitate cultures. (**b**) Bacteriophage immobilized to magnetic nanoparticles were added to the sample and incubated shaking for 10 min at 37 °C. (**c**) *E. coli* cells in the sample were captured by the bacteriophage immobilized nanoparticles. (**d**) A magnet was used to concentrate the bacteria-bacteriophage nanoparticle complex allowing the supernatant removal. (**e**) After 3 h of incubation (shaking at 37 °C) to allow for luciferase reporter enzyme expression and bacteria lysis to occur, samples were filtered through a 96 well filter plate to capture the luciferase reporter enzyme. (**f**) Luciferase substrate was added to each well and incubated at room temperature for 10 min for reaction to occur. (**g**) Luminescent signal was read in a spectrophotometer.

**Figure 4:**
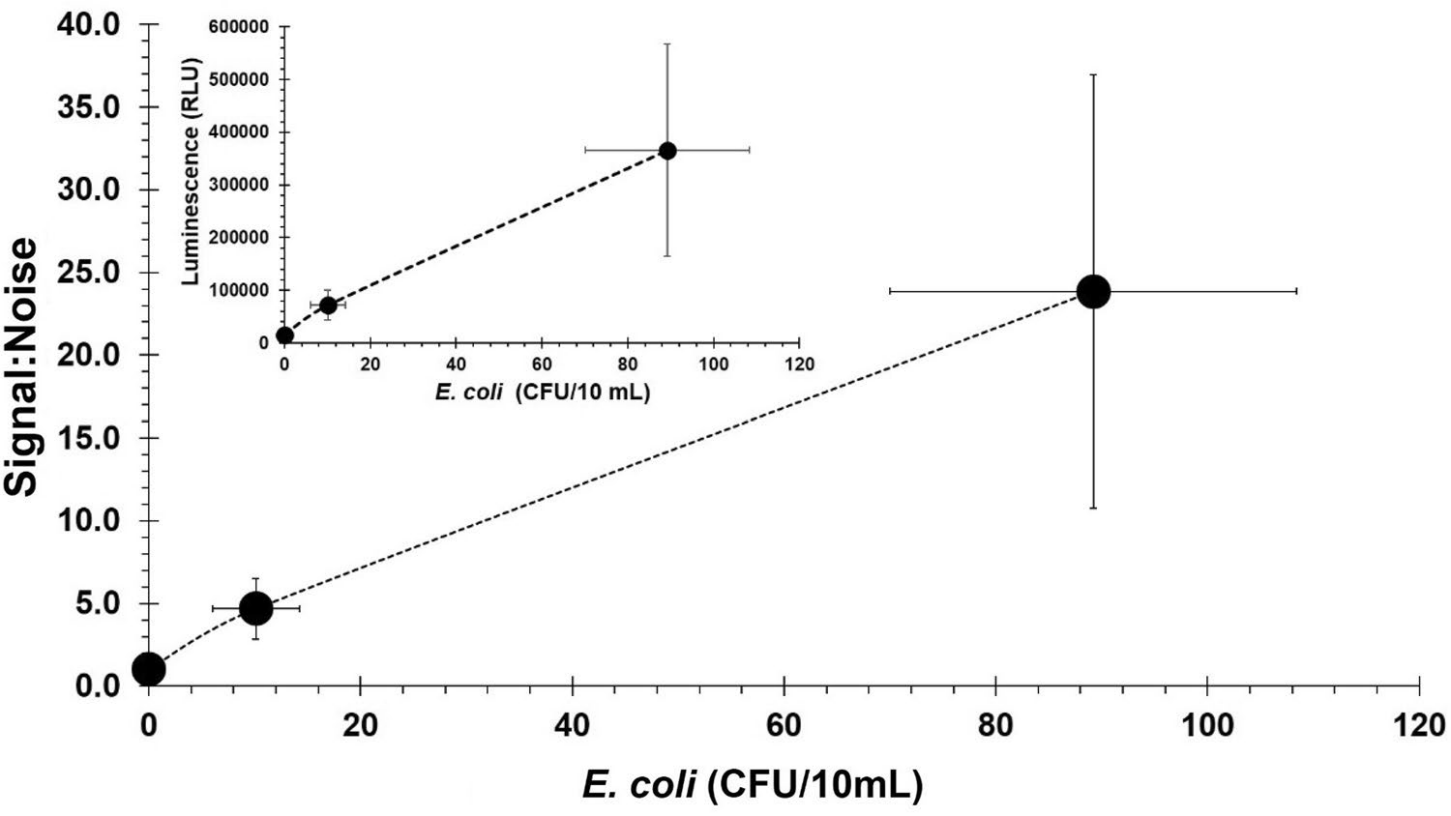
*E. coli* detection assay in 10 mL of water. Use of NRGP56 phage particles for separation and detection of varying concentrations of *E. coli* spiked in 10 mL sterile tap water samples. Error bars represent standard deviations from biological triplicates.

The assay workflow for target bacteria resuscitation, separation, concentration, and detection using NRGP56 and NRGP28 phages immobilized to magnetic nanoparticles is depicted in Fig. 3. The assay was initially conducted on ECOR13, an *E. coli* environmental isolate used in previous detection assays^62,63^, in a 10 mL water sample. Standard plate counts of the inoculated water samples were performed alongside the phage-based detection assay for comparison. The assay’s signal increased proportionally with ECOR13 concentration. The assay resulted in a 4.68 ± 1.82 signal:noise for 10 ± 4 CFU and 23.85 ± 13.11 signal:noise for 89 ± 19 CFU as seen in Fig. 4. The limit of detection (LOD) for ECOR13 in 10 mL of water using the NRGP56 phage assay was calculated to be approximately 1 CFU/10 mL.

**Figure 5:**
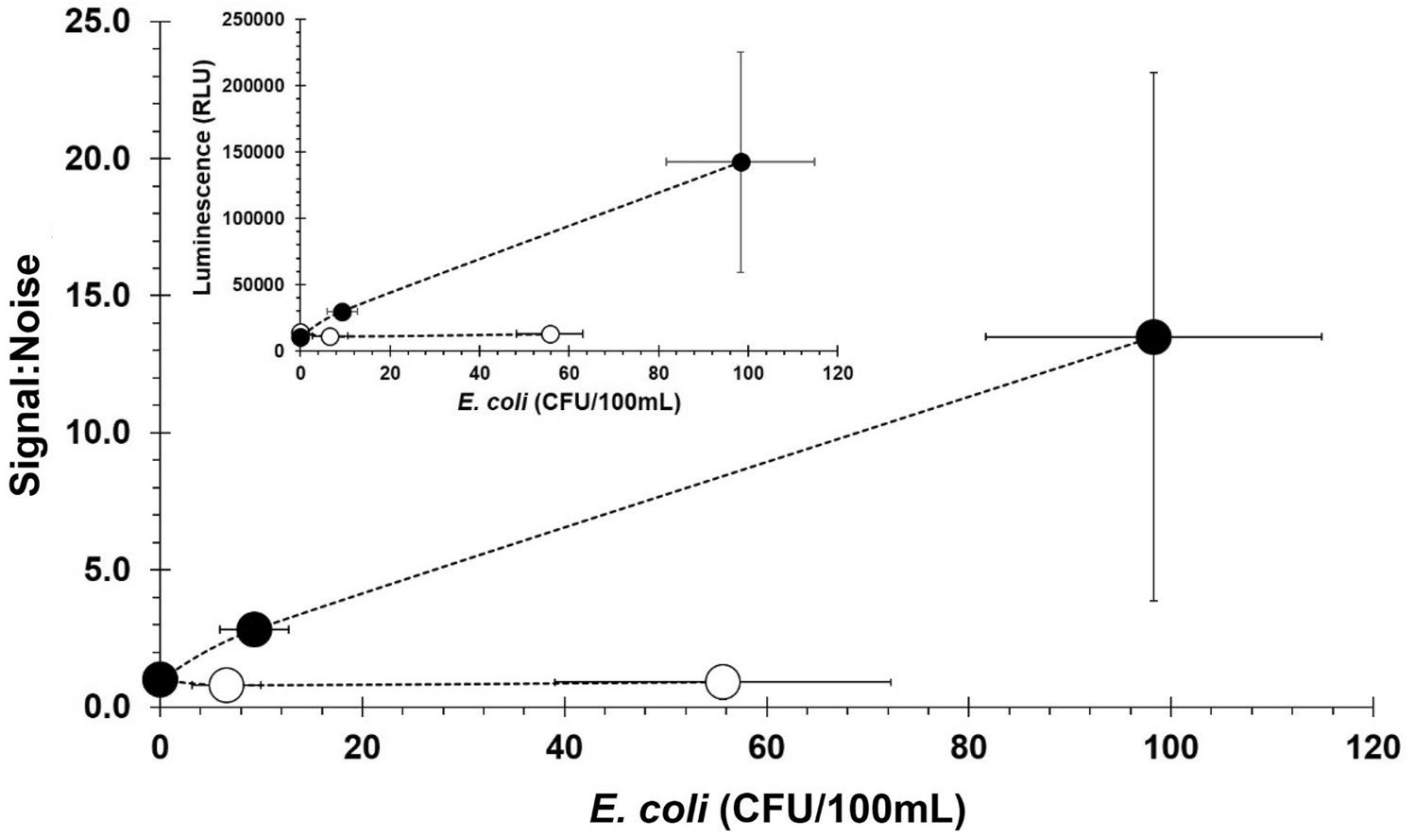
*E. coli* detection assay in 100 mL of water. Comparison of NRGP56 phage particles (●) to NRGP28 phage particles (○) for separation and detection of varying concentrations of *E. coli* spiked in 100 mL sterile tap water samples. Error bars represent standard deviations from biological triplicates.

To assess real world applications, the NRGP56 phage assay was scaled-up to be used on 100 mL water samples as required by the United States Environmental Protection Agency for drinking water. The assay’s signal increase remained proportional with *E. coli* ECOR13 concentration. The assay resulted in a 2.83 ± 0.47 signal:noise for 9 ± 3 CFU and 13.50 ± 7.87 signal:noise for 98 ± 17 CFU as seen in Fig. 5. The limit of detection (LOD) for *E. coli* ECOR13 in 100 mL of water using the NRGP56 phage assay was calculated to be approximately 5 CFU/100 mL.

During immobilization of phages to the magnetic nanoparticles’ assays described above, results indicated majority of the NRGP28 control phages were remaining immobilized to the particles even after washing. To determine if wild type Hoc alone is sufficient to provide stable immobilization that can be maintained during practical use, an NRGP28 phage detection assay was performed in 100 mL water samples. The assay’s signal change was not proportional with ECOR13 concentration change. As can be seen in Fig. 5, all the variable ECOR13 concentrations had slightly lower signal:noise than the 0 CFU control. The limit of detection (LOD) for ECOR13 in 100 mL of water using the NRGP28 phage assay was calculated to be 9 × 10^6^ CFU.

A one-way ANOVA was performed within each of the 3 trials on the 3 means of the varying *E. coli* concentrations. The NRGP56 phage detection assays produced extremely sensitive LODs (~1 and ~5 CFU for 10 and 100 mL, respectively), with significance against the control (Fig. S1). The poor performance of the NRGP28 phage detection assay suggests that the electrostatic adsorption interactions that were able to immobilize NRGp28 phages to the particles are not strong enough to keep the phages immobilized during functional use in our detection assay. This could be due to the binding of the phage to the bacteria being a stronger interaction than the electrostatic adsorption to the particles. During the T4 phage infection, phage short tail fibers bind irreversibly to their receptors on the bacteria’s outer membrane and phage tail tube components fuse with the bacteria’s cytoplasmic membrane^64^. When the magnet separated the particles from the matrix to concentrate the target bacteria, the phages were likely left behind on the bacteria instead of remaining attached to both the particles and bacteria. This suggests the necessity of the mSA modification to Hoc for the execution of this sensitive bacteria detection assay.

## Conculsions

This work highlights a method to efficiently conjugate bacteriophages to biotinylated surfaces using a commercially scalable and low-cost method. The genetically modified phages with a novel monomeric streptavidin capsid modification allowed for targeted immobilization to biotin functionalized particles.

While previous reported methods have chemically modified phages to biotin for conjugation to streptavidin-modified surfaces^65–68^, we report a method which results in mSA modified phages during propagation. The mSA has a reported Kd of 2.8 nM^43^ and approximately 155 are displayed per phage capsid in a repeating pattern. Functionalization of phages via targeting of primary amines or carboxylic acids can result in loss of phage infectivity due to inadvertent conjugation to proteinaceous tail fibers.

Other functionalization methods include sodium periodate oxidation then oxime formation^69^ and diazo linkage then copper catalyzed azide-alkyne cycloaddition^70^. These reactions can also react with non-target exposed compatible functional groups, risking unintentional modification of phage components that can interfere with proper phage function. More site-specific chemical conjugation methods relying on an initial genetic unnatural amino acid incorporation have been used to modify phages with biotin^71–74^ but suffer from the complexity and inefficiency of the unnatural amino acid incorporation process^75,76^. Phages have been genetically modified with biotin carboxyl carrier proteins, but this requires an extra step for the proteins to be biotinylated by a biotin ligase enzyme within the system for phage production^36,77–80^. Additionally, this would require streptavidin modification of the immobilization surface which reduces commercial viability due to the cost. In our study, once the initial site-specific genomic modification was performed, the NRGP56 can be propagated using the same machinery, methods, and workflows as wild type phages allowing for an overall low-cost modification. To our knowledge, this is the first phage genetically modified to express a monomeric streptavidin on its capsid.

### Supplementary Information

Supplementary Information 1.

Supplementary Information 2.

## Data Availability

The datasets generated/ analyzed as well as biological samples synthesized during the current study are available from the corresponding author (snugen@cornell.edu) on reasonable request.
