# Supplementary material for: Monomeric streptavidin phage display allows efficient immobilization of bacteriophages on magnetic particles for the capture, separation, and detection of bacteria
Source: Sci Rep. 2023 Sep 27;13:16207. doi: 10.1038/s41598-023-42626-9 (PMC10533843; doi:10.1038/s41598-023-42626-9)
Supplement: Supplementary file 1 — Supplementary Information 1. [file 41598_2023_42626_MOESM1_ESM.pdf]

Monomeric Streptavidin Phage Display Allows Efficient Immobilization of Bacteriophages on Magnetic Particles for the Capture, Separation, and Detection of Bacteria

Caitlin M. Carmody and Sam R. Nugen

Figure S1: Detection of *E. coli* in 10 mL of water

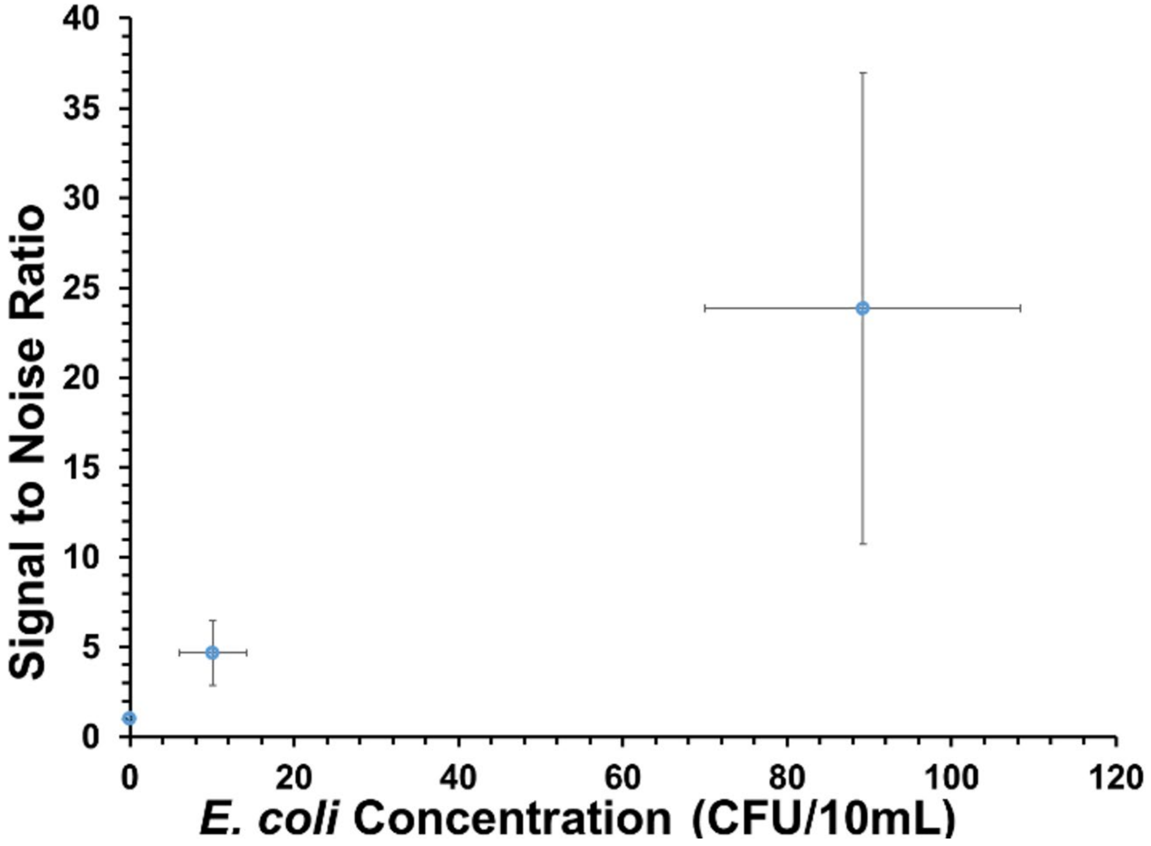

E. coli detection assay in 10 mL of water. NRGp56 phage particles used for separation and detection of varying concentrations of *E. coli* spiked in 10 mL sterile tap water samples. Luminescence normalized to 0 CFU control. Error bars represent standard deviation from three biological replicates. Vertical error bars represent the standard deviation from the phage assay and the horizontal error bars represent the standard deviation from plate counts.

Table S1: Primers used for experiments

NRG primer number	*NRG primer name*	Sequence	Purpose
2265	*cc-hocbbF*	atgatggtgatg**cataagttatccttattttaatgttacgaaagaag**	Anneals to region of homology upstream of *hoc* to fuse to *mSA*
2266	*cc-hocbbR*	gcgtctggatcc**ccaggcggcatgacttttac**	Anneals to *hoc* to fuse *mSA* to the N-terminus of *hoc*
2267	*cc-msaF*	tcatgccgcctgg**ggatccagacgccgcaga**	Anneals to *mSA* to fuse *mSA* to the region of homology upstream of *hoc*
2268	*cc-msaR*	aggataacttatg**catcaccatcatcaccacagc**	Anneals to *mSA* to fuse *mSA* to the region of homology upstream of *hoc*
1310	*cc-hoc-down-F*	acgtgagttttc**aactaattcctttttaatcaaagaaggattatc**	Anneals to region of homology downstream of *hoc* to add to pCRISPR
1494	*cc-hocup-PAM-F*	agaagttgatgtt**caagaatcgcgtaatggatac**	Anneals to *hoc* to change gRNA 16 PAM site to impart resistance to cleavage
1495	*cc-hocdown-PAM-R*	tacgcgattcttgaacatc**aacttctggatagtctttc**	Anneals to *hoc* to change gRNA 16 PAM site to impart resistance to cleavage
1518	*cc-CBM-HOC-FixHifi-F*	agttatatcaactg**taaaagtcatgccgcctg**	Anneals to *hoc* to change gRNA 20 codons to impart resistance to cleavage
1519	*cc-CBM-HOC-FixHifi-R*	gcatgacttttac**agttgatataactcctaaaacacc**	Anneals to *hoc* to change gRNA 20 codons to impart resistance to cleavage
1311	*cc-hoc-down-R*	tgcactgtcgga**tcataaggggcttcggcc**	Anneals to region of homology downstream of *hoc* to fuse to *hoc*
1316	*cc-hoc-up-R*	gctcagtggaacc**cagttgccatatctgaag**	Anneals to region of homology upstream of *hoc* to fuse to pCRISPR
1270	*CC-hoc-g16F*	aaac**tgtatccattacgcgattct**g	gRNA16 with overhangs to anneal to pCRISPR
1271	*CC-hoc-g16R*	aaaac**agaatcgcgtaatggataca**	gRNA16 with overhang to anneal to pCRISPR
1278	*CC-hoc-g20F*	aaac**gtttcatcaataacccctgt**g	gRNA20 with overhangs to anneal to pCRISPR
1279	*CC-hoc-g20R*	**aaaac**acaggggttattgatgaaac	gRNA20 with overhang to anneal to pCRISPR
1308	*cc-pCRISPR-lin-F*	atatggcaactg**ggttccactgagcgtcagac**	Anneals to pCRISPR and fuses to region of homology upstream of *hoc*
1309	*cc-pCRISPR-lin-R*	aaaggaattagttg**aaaactcacgttaagggattttg**	Anneals to pCRISPR and fuses to region of homology downstream of *hoc*

Bold portion of sequences are the portion of the primer that anneals to the DNA template. Non-bold parts of the primer are the 5' overhangs incorporated for Gibson Assembly Cloning. For gRNA the bold is the gRNA sequence and non-bold is the portion that ligates to the digested Golden Gate Cloning site.

Figure S2: mSA-Hoc Donor Region Sequence

(ROH upstream – his tag – mSA – linker – hoc – ROH Down)

CAGTTGCCATATCTGAAGCGAGGATTAAATCTATATCCATCGGCCTCAAACGAAAAGAAATCTCTTAATTCGTGGA
ACGTGCTCTCTTCACAATCGATGCGTACATGACTGAAGTCGTGAAAATGTACTTTAATATCCATAATTATGCCTTAC
TAAATTTGCCTTTAGAATCTCTTTTCATGAGACGACCTTTAATAAATCCGTCGGGAATAATACCGTCTGGTTGTATTA
ATTTATTTATTGCGCCATTATTGACCCAAAAAGTTCCTGTGGTAGTCGGTTTGACTTTACATCCTTTTCTGGACTTTC
TATTAGGATGAACCATCCCTTTTACAAATCCTTCTGGAACAAGTTCTCCAGGTTTAATAAAAATATTTTTAGTTCCAT
TAGTATAACAAGTTTTACCTAATACCGTGCCCGGTGAGTTTTCAAATCTTTTAGCGGAAGATTCTTTCATCTTTGCTA
TAACATCTGTAGTCATAACAATTCCACCAATTCCACCAGGTTTCATATTATAATAATTTTTGCTTTTTATTAGTTCAGG
AGTTATAATTTCTTCTTCATACATGTACGCTTCTTCGGAAGTTTTAAACTCTTTTAGTATTGTTCTAGAGAAATTGTTT
TCACCATATTTCTTTATAGCCTGCTGAATTGCCTTACCGGAACCAAGGTAACCATCATTCAAGTCATCAGTAGAGTG
CTTTCCTATATACTTTTTACCATTTATTAGATTTGTTGTTTCATATACAAAGTGGTACATACTATTTTCCGAGTAATAA
ATATATCTATATTTATACTGAGGAAATATTATGATAGATAAAGATTATATTGCAGAGCTGAAGGCTCTTGATGATA
ACAAAGAAGCTAAAGCTAAATTAGCTGAATATGCTGAACAGTTTGGTATAAAGGTCAAAAAGAATAAATCTTTTGA
TAATATCGTTGTTGATATTGAAGAAGCCCTCCAGAAGCTCGCTAGTGAACCTATGCCAGAGACTGATGGGTTATCT
ATTAAAGACTTAATTGATGCTGCTGATGCCGCAGAGGGATTAAAATATGACGATGAAGAAGTCAATCCAGAAGCA
GCACTTCTGATTGATTCTCCGATTAAATCTGACATTAAAATTGAAGTAGTAGAAACGGATAAAATTCCTGAAAATAC
CGATGTTTTGATTGAAGATACTCCTTTTGTTGAAGAAAAAATTCGAACAGGCTGTAGCTGAGATTATTGAATCTGAA
AAGCCGTCTGTATTTACTCTTCCGGAAAACTTTAGTCCGAATCTTCAACTGATTGGAAAAAATCTAGGATTCTGTAC
TGTTCCTTGGTGGATTTATCAATGGATTGCTGAAACTCCTGATTGGAAATCTCACCCAACTAGTTTTGAACATGCGT
CAGCACACCAAACTTTATTTAGCTTAATTTATTACATTAATCGCGATGGATCAGTTTTAATTCGTGAAACACGCAAC
TCTTCTTTCGTAACATTAAAATAAGGATAACTTATGCATCACCATCATCACCACAGCCAGGATCTGGCTAGCGCGGA
AGCGGGTATCACCGGCACGTGGTACAACCAGTCTGGTTCTACCTTCACCGTTACCGCGGGTGCGGACGGTAACCT
GACCGGTCAGTACGAAAACCGTGCGCAGGGCACTGGTTGCCAGAACTCTCCGTACACCCTGACCGGTCGTTACAA
CGGTACCAAACTGGAATGGCGTGTTGAATGGAACAACTCTACCGAAAACTGCCACTCTCGTACCGAATGGCGTGG
TCAGTACCAGGGTGGTGCGGAAGCGCGTATCAACACCCAGTGGAACCTGACCTACGAAGGTGGTTCTGGTCCGGC
GACCGAACAGGGTCAGGACACCTTCACCAAAGTTAAACCGTCTGCGGCGTCTGGATCCCCAGGCGGCATGACTTT
TACAGTTGATATAACTCCTAAAACACCTACTGGTGTAATAGACGAGACTAAGCAGTTTACTGCTACACCCAGTGGT
CAAACTGGAGGCGGAACTATTACATATGCTTGGAGCGTAGATAATGTTCCACAAGATGGAGCTGAAGCAACTTTT
AGTTATGTACTAAAAGGACCTGCCGGTCAAAAGACTATTAAAGTAGTTGCAACAAATACACTTTCTGAAGGAGGCC
CGGAAACGGCTGAAGCGACAACAACTATCACAGTTAAAAATAAGACACAGACGACTACCTTAGCCGTAACTCCTG
CTAGTCCTGCGGCTGGAGTGATTGGAACCCCAGTTCAATTTACTGCTGCCTTAGCTTCTCAACCTGATGGAGCATCT
GCTACGTATCAGTGGTATGTAGATGATTCACAAGTTGGTGGAGAAACTAACTCTACATTTAGCTATACTCCAACTA
CAAGTGGAGTAAAAAGAATTAAATGCGTAGCCCAAGTAACCGCGACAGATTATGATGCACTAAGCGTTACTTCTA
ATGAAGTATCATTAACGGTTAATAAGAAGACAATGAATCCACAGGTTACATTGACTCCTCCTTCTATTAATGTTCAG
CAAGATGCTTCGGCTACATTTACGGCTAATGTTACGGGTGCTCCAGAAGAAGCACAAATTACTTACTCATGGAAGA
AAGATTCTTCTCCTGTAGAAGGGTCAACTAACGTATATACTGTCGATACCTCATCTGTTGGAAGTCAAACTATTGAA
GTTACTGCAACTGTTACTGCTGCAGATTATAACCCTGTAACCGTTACCAAAACTGGTAATGTAACAGTCACGGCTA
AAGTTGCTCCAGAACCAGAAGGTGAATTACCTTATGTTCATCCTCTTCCACACCGTAGCTCAGCTTACATCTGGTGC
GGTTGGTGGGTTATGGATGAAATCCAAAAAATGACCGAAGAAGGTAAAGATTGGAAAACTGACGACCCAGATAG
TAAATATTACCTGCATCGTTACACTCTCCAGAAGATGATGAAAGACTATCCAGAAGTTGATGTTCAAGAATCGCGT
AATGGATACATCATTCATAAAACTGCTTTAGAAACTGGTATCATCTATACCTATCCATAATCATAAGGGGCTTCGGC

CCCTTTCTTCATTTTGAAAGCACACAAAACACAATCAGAAAATGATGTATATAATGGCACCAACTCGATAACATGA
GATTGATTATGAGAACTGAGGTTGTGGTGTTTACTCTTCATGAGTCTGGAAAGTCATTCATTGAAATTGCTCGTGA
ATTAAACTTACAGGCAAAAGAAGTGGCTGTATTATGGGCTCGAGAAAAAGTTGTCTATAGAAAAAGACATATCAA
TAAAAAGGTGAAAAATGGAACAGTATGATCTTTATGAAAATGAATCTTTTGCTAATCAATTACGCGAAAAAGCATT
AAAAAGTAAACAGTTTAAGCTAGAGTGTTTTATTAAAGATTTTTCAGAACTTGCTAATAAAGCAGCTGAACAAGGT
AAAACACATTTTAATTATTATTGTATTGCTCGTGATAAATTGATTACAGAAGAAATTGGTGATTGGCTGAGAAAAG
AAGGATTCAGCTTTAAAGTCAATAGTGATCAGCGTGATGGTGATTGGTTAGAAATTACATTTTGAGGATTAATTAT
GTTTAAAAAGTATAGCAGTCTTGAAAATCATTACAACTCTAAATTTATTGAAAAACTTTATAGCTTGGGATTGACTG
GTGGGGAGTGGGTAGCTCGTGAAAAGATTCACGGCACAAATTTCTCATTGATTATTGAGCGTGATAAAGTGACTT
GCGCTAAACGCACTGGACCGATTCTTCCTGCTGAAGATTTCTTTGGGTATGAAATTATTTTGAAGAATTATGCTGAT
TCCATTAAAGCTGTACAAGATATTATGGAAACCTCAGCGGTTGTATCTTATCAAGTCTTTGGCGAATTCGCTGGACC
TGGCATTCAGAAGAATGTTGATTATTGTGATAAAGATTTTTATGTATTTGACATTATTGTTACTACAGAAAGCGGTG
ATGTGACTTATGTAGATGATTATATGATGGAATCATTCTGTAATACATTTAAATTTAAAATGGCTCCACTTTTAGGT
CGCGGTAAATTTGAAGAGCTTATTAAATTGCCAAATGATTTAGATTCTGTCGTCCAAGATTATAATTTTACAGTAGA
CCATGCTGGATTAGTTGATGCAAATAAATGCGTTTGGAATGCCGAAGCAAAAGGCGAAGTATTTACTGCTGAAGG
ATATGTATTGAAACCTTGTTATCCTTCTTGGCTTCGTAATGGAAATCGTGTAGCGATTAAATGCAAGAACTCTAAAT
TTAGTGAAAAGAAAAAGTCTGATAAGCCTATTAAAGCTAAAGTTGAGCTATCAGAAGCTGATAACAAATTGGTGG
GAATTTTAGCTTGTTACGTTACACTGAACCGCGTAAATAACGTTATTTCTAAAATTGGCGAAATTGGTCCAAAGGAT
TTTGGAAAGGTGATGGGGCTAACTGTTCAAGATATTTTGGAAGAAACTTCTCGTGAAGGTATTACTCTAACTCAAG
CAGATAATCCTTCTTTGATTAAAAAGGAATTAGTTG

Table S2: 10ml mSA-Hoc T4 phage *E. coli* Detection Assay

**Plate Count Raw Data**	*E. coli* 1	*E. coli* 1	*E. coli* 1	*E. coli* 2	*E. coli* 2	*E. coli* 2	*E. coli* 3	*E. coli* 3	*E. coli* 3
-8 Dilution (CFU)	13	4	8	16	8	6	16	9	12
-7 Dilution (CFU)	68	85	66	69	123	102	112	83	95

Table S3: Raw data for 10ml mSA-Hoc T4 phage *E. coli* Detection Assay

**RLU Raw Data**	0 CFU (RLU)	-8 Dilution (RLU)	-7 Dilution (RLU)
E. coli 1	11196	92737	169689
E. coli 2	14852	32168	285637
E. coli 3	19979	90314	642292

Table S4: Statistics for 10ml mSA-Hoc T4 phage *E. coli* Detection Assay

**One-way ANOVA of RLU Data**	Sum of Squares	Df	Mean squares	F Value	P-value	F Critical
Between Groups	27.02491	2	13.51246	4.023984	0.077914	5.143253
Within Groups	20.14788	6	3.35798			
Total	47.17279	8				

Table S5: 100ml mSA-Hoc T4 phage *E. coli* Detection Assay

**Plate Count Raw Data**	*E. coli* 1	*E. coli* 1	*E. coli* 1	*E. coli* 2	*E. coli* 2	*E. coli* 2	*E. coli* 3	*E. coli* 3	*E. coli* 3
-8 Dilution (CFU)	16	10	7	8	12	11	9	3	8
-7 Dilution (CFU)	106	108	107	89	102	95	94	124	60

Table S6: Raw data for 100ml mSA-Hoc T4 phage *E. coli* Detection Assay

**RLU Raw Data**	0 CFU (RLU)	-8 Dilution (RLU)	-7 Dilution (RLU)
E. coli 1	7746	29846	81329
E. coli 2	14990	23761	260022
E. coli 3	8940	35905	86314

Table S7: Statistics for 100ml mSA-Hoc T4 phage *E. coli* Detection Assay

**One-way ANOVA of RLU Data**	Sum of Squares	Df	Mean squares	F Value	P-value	F Critical
Between Groups	273.5781	2	136.789	4.396059	0.066736	5.143253
Within Groups	186.6977	6	31.11628			
Total	460.2758	8				

Table S8: 100ml WT-Hoc T4 phage *E. coli* Detection Assay

**Plate Count Raw Data**	*E. coli* 1	*E. coli* 1	*E. coli* 1	*E. coli* 2	*E. coli* 2	*E. coli* 2	*E. coli* 3	*E. coli* 3	*E. coli* 3
-8 Dilution (CFU)	5	7	6	3	8	6	1	16	7
-7 Dilution (CFU)	43	54	48	56	49	64	67	61	59

Table S9: Raw data for 100ml WT-Hoc T4 phage *E. coli* Detection Assay

**RLU Raw Data**	0 CFU (RLU)	-8 Dilution (RLU)	-7 Dilution (RLU)
E. coli 1	5600	13206	8275
E. coli 2	20014	4059	7308
E. coli 3	16968	16554	23113

Table S10: Statistics for 100ml WT-Hoc T4 phage *E. coli* Detection Assay

**One-way ANOVA of RLU Data**	Sum of Squares	Df	Mean squares	F Value	P-value	F Critical
Between Groups	0.063796	2	0.031898	0.108293	0.899079	5.143253
Within Groups	1.767304	6	0.294551			
Total	1.831099	8				
